# Liver-Specific Inactivation of the Proprotein Convertase FURIN Leads to Increased Hepatocellular Carcinoma Growth

**DOI:** 10.1155/2015/148651

**Published:** 2015-06-08

**Authors:** Jeroen Declercq, Bas Brouwers, Vincent P. E. G. Pruniau, Pieter Stijnen, Krizia Tuand, Sandra Meulemans, Annik Prat, Nabil G. Seidah, Abdel-Majid Khatib, John W. M. Creemers

**Affiliations:** ^1^Laboratory for Biochemical Neuroendocrinology, Department of Human Genetics, KU Leuven, Herestraat 49, 3000 Leuven, Belgium; ^2^Laboratory of Biochemical Neuroendocrinology, Institut de Recherches Cliniques de Montréal, Montreal, QC, Canada H2W 1R7; ^3^Angiogenesis Laboratory, Université Bordeaux I, 33400 Talence, France; ^4^INSERM, UMR 1029, 33400 Talence, France

## Abstract

Proprotein convertases are subtilisin-like serine endoproteases that cleave and hence activate a variety of proproteins, including growth factors, receptors, metalloproteases, and extracellular matrix proteins. Therefore, it has been suggested that inhibition of the ubiquitously expressed proprotein convertase FURIN might be a good therapeutic strategy for several tumor types. Whether this is also the case for hepatocellular carcinoma (HCC) is currently not clear. In a mouse model for HCC expression of *Furin* was not altered in the tumors, while those of PC7, PC5/6, and PACE4 significantly decreased, at least at some time points. To investigate the impact of *Furin* inhibition on the development and progression of HCC in this model, *Furin* was genetically ablated in the liver. *Furin* inactivation resulted in an increased tumor mass after 5 weeks. This was not caused by decreased apoptosis, since no differences in the apoptosis index could be observed. However, it could at least partially be explained by increased hepatocyte proliferation at 5 weeks. The tumors of the *Furin* knockout mice were histologically similar to those in wild type mice. In conclusion, liver-specific *Furin* inhibition in HCC enhances the tumor formation and will not be a good therapeutic strategy for this tumor type.

## 1. Introduction

Furin is an endoprotease that belongs to the seven-member family of subtilisin-like proprotein convertases (PCs) [1, 2]. The other family members are PC1/3, PC2, PC4, PC5/6, PC7, and PACE4. Their physiological role is to cleave a variety of precursor proteins (proproteins), carboxy-terminal of specific basic amino acid motifs. Cleavage is usually needed for activation of the proteins, although exceptions exist in which cleavage results in inactivation, modified or even opposite activity. Precursor proteins include growth and differentiation factors, receptors, adhesion molecules, and enzymes like matrix metalloproteinases (MMPs). They play important roles at different stages of tumor development, progression, vascularization, and metastasis. Therefore, it is not surprising that the aggressiveness of tumors has been correlated with increased* PC* expression, including breast, ovary, brain, skin, prostate, and lung cancer [3–10]. In particular, the broadly expressed PCs Furin and PACE4 have frequently been found to be highly expressed in tumors. Nevertheless, upregulation of the neuroendocrine specific members PC1/3 and PC2 has also been reported in, for example, lung tumors [5, 11].

Furthermore, it has been shown that inhibition, knockdown, and genetic ablation of Furin reduces tumorigenesis in various human cancer cell lines [12]. For example, FURIN inhibition in squamous cell carcinoma cell lines resulted in a decreased proliferation, reduced anchorage-independent growth in soft agar assays, and inhibited* in vivo *tumorigenicity and invasion in nude mice [13]. In contrast,* Furin *overexpression in these cell lines resulted in the opposite phenotype and increased their proliferation and invasiveness [14].

Similarly, transgenic mice overexpressing* Furin *display enhanced skin tumor formation [15], whereas we previously demonstrated that genetic ablation of* Furin* in salivary gland tumors reduces the tumor formation [16]. Indeed, the development and progression of* PLAG1*-induced pleomorphic adenomas of the salivary glands was inhibited with a simultaneous genetic ablation of* Furin*. The delayed tumor onset after genetic ablation of* Furin* in the salivary glands could be explained by the partially impaired processing of the insulin-like growth factor 1 receptor (IGF1R) in the salivary glands. In this way it interferes with IGF signaling, a major tumorigenic pathway involved in* PLAG1*-induced salivary gland tumors [17, 18].

These* in vitro* and* in vivo* lines of evidence suggest that FURIN inhibition might be a possible anticancer therapy [19]. Therefore, FURIN inhibitors have been generated [20, 21]. For instance, decanoyl-Arg-Val-Lys-Arg-chloromethylketone (decRVKR-CMK) and *α*1-antitrypsin Portland (*α*1-PDX) could decrease cell proliferation rate and invasiveness of cancer cell lines [6, 14]. However, most FURIN inhibitors generated so far are not specific for FURIN but also inhibit other members of the proprotein convertases. Nevertheless, in some cases, administration of specific FURIN inhibitors might be required. It has been described that genetic ablation of other proprotein convertases such as PC5/6 enhances tumor formation [22]. For example enterocyte-restricted ablation of PC5/6 in the APC^Min/+^ mouse model resulted in a significantly higher tumor number in the small intestines, indicating that PC5/6 protects against tumorigenesis. For this reason specific FURIN inhibitors rather than general proprotein convertase inhibitors may be needed to obtain therapeutic benefit. Recently, specific FURIN inhibitors were generated such as nanobodies against catalytically active FURIN [23] and an engineered *α*1-antitrypsin variant [24]. Those inhibitors will now have to be validated on cancer cell lines. Recently, bifunctional shRNA against* Furin *was used in patients with advanced cancer in a phase 1 clinical trial of FANG, a bi-shRNAiFurin/GMCSF DNA/Autologous tumor vaccine [25]. This treatment was safe and induced an immune response that correlated with a longer overall survival.

With an estimated 750,000 new cases worldwide in 2008, hepatocellular carcinoma (HCC) is the third most common cause of cancer death [26]. Despite advances in the treatment and early diagnosis of HCC more than 70% of HCC patients will not benefit from the treatment because of the advanced stage of the disease at the moment of diagnosis. Therefore, the prognosis of HCC patients is generally very poor with a 5-year survival rate less than 5%. New therapeutic strategies to treat this disease are thus urgently required. FURIN inhibition might be such a possible strategy. However, so far the role of FURIN in hepatocellular carcinoma (HCC) is not yet clear.* FURIN *overexpression in HepG2 cells increased the metastasis index and tumor size in animal models [27]. This suggests that FURIN serves as an anticancer target in HCC. Nevertheless, the clinical relevance of this finding was challenged since a tumor/nontumor ratio of* Furin* of more than 3.5 in HCC tissue predicted a better postoperative disease-free survival in a postoperative survival analysis of HCC patients [28]. Furthermore, Huh7 cells overexpressing* FURIN* displayed a reduced tumor growth in subcutaneous xenograft experiments, which could be reversed by administration of synthetic FURIN inhibitors [28]. Therefore, it is still unclear whether or not* FURIN* inhibition might be a good strategy for HCC. In this study we have investigated the therapeutic relevance of FURIN inhibition for the treatment of HCC via genetic ablation of* Furin *in the liver in a mouse model for HCC.

## 2. Materials and Methods

### 2.1. Mouse Models

All animal experiments were approved by the ethical committee of the KU Leuven and were performed in a C57Bl/6J background.

Fur^fl/fl^ mice were generated as described previously [29]. In brief, exon 2 was flanked by loxP sites. This allowed the conditional removal of exon 2, which contains the translation initiation site, the signal peptide and part of the pro-domain, leading to the inactivation of the* Furin *gene [29].

The ASV-B mice were used as a tumor model for HCC (a kind gift of Dr. Evelyne Dupuy [30]). HCC was induced by the expression of the Simian vacuolating virus 40 (SV40)-T antigen under the control of the anti-thrombin III promoter. The transgene was integrated on the Y-chromosome. As a consequence male mice developed hepatic tumors, while female mice remained tumor free.

B6·Cg-Tg(Alb-cre)21Mgn/J mice (The Jackson Laboratory stock no 003574), here abbreviated as Alb-Cre mice, were used to conditionally inactivate* Furin* in the liver [31]. In this model Cre is expressed in the liver, driven by the albumin promoter.

The ASV-B mice were intercrossed with Fur^fl/fl^ mice for two generations to obtain ASV-B^+/−^ Alb-Cre^−/−^  Fur^fl/fl^ mice. In parallel the Alb-cre mice were intercrossed with Fur^fl/fl^ mice for two generations to obtain ASV-B^−/−^ Alb-Cre^+/−^  Fur^fl/fl^ mice. Finally, ASV-B^+/−^ Alb-Cre^−/−^  Fur^fl/fl^ mice were intercrossed with ASV-B^−/−^ Alb-Cre^+/−^  Fur^fl/fl^ mice to obtain ASV-B^+/−^ Alb-Cre^+/−^  Fur^fl/fl^ (tumor bearing male mice, in which* Furin* is inactivated in the liver), ASV-B^+/−^ Alb-Cre^−/−^  Fur^fl/fl^ (tumor bearing male mice, in which* Furin* is present in the liver), ASV-B^−/−^ Alb-Cre^+/−^  Fur^fl/fl^ (female mice without tumors, in which* Furin *is inactivated in the liver) and ASV-B^−/−^ Alb-Cre^−/−^  Fur^fl/fl^ mice (female mice without tumors, in which* Furin* is present in the liver). Those offspring mice were used for the experiments.

Mice were genotyped by PCR analysis of tail DNA using the primes 5′ GCTGTATTTATTCCGGAGAC 3′ and 5′ GTAGTTAGGAGCACATACTG 3′ to distinguish between* Furin* floxed and wild type alleles and 5′ CCTGTTTTGCACGTTCACCG 3′ and 5′ ATGCTTCTGTCCGTTTGCCG 3′ to detect the presence of the Cre recombinase. Sexing of the mice was sufficient to determine the presence of the SV40-T transgene.

### 2.2. Quantitative Real-Time PCR

Total RNA was isolated from the liver and liver tumors using the Nucleospin RNA midi (Macherey Nagel, Düren, Germany) according to the manufacturer's protocol. First strand cDNA was synthesized using iScript cDNA synthesis kit (Bio-Rad, Hercules, CA). Primers were designed with the ProbeFinder software (Roche, Basel, Switzerland, listed in Table S1 in supplementary materials available online at http://dx.doi.org/10.1155/2015/148651). Quantitative real-time PCR (qRT-PCR) was performed in triplicate with MyIQ Single Color Real-Time PCR Detection System (Bio-Rad) using SYBR Green. Samples were normalized to glyceraldehyde 3-phosphate dehydrogenase (*Gapdh*). Data were analysed using the Livak method [32].

### 2.3. Histological Analysis

Mouse livers were fixed in 4% formaldehyde in PBS, embedded in paraffin and sectioned at 5 *μ*m thickness. Sections were stained with Gill's haematoxylin and eosin or with sirius red staining according to standard procedures.

### 2.4. Quantification of the Proliferation and Apoptosis Index

Ki67 staining (proliferation) and active-caspase3 staining (apoptosis) were performed on 4 liver sections per tumor. Those areas were randomly selected from the slides based on the DAPI staining, to compensate for the possibly uneven distribution of the apoptotic/proliferating cells within the tumor mass. Rehydrated sections were heated for 20 minutes in Target Retrieval Solution (pH 6.1, Dako). After blocking with 20% normal goat serum (Dako) in PBS, slides were incubated overnight at 4°C with polyclonal rabbit anti-Ki67 antibody (1 : 500, Abcam, ab15580) or rabbit anti-active caspase3 antibody (1 : 400, Cell Signaling Technology, 9664S) each diluted in Dako Antibody Diluent. As secondary antibody the Alexa fluor 488 goat anti-rabbit antibody was used for 1 hour at room temperature and nuclei were stained with DAPI (dilution 1/10000, Dako). Images were taken using the Leica DMRB Fluorescence microscope. Ki67, active caspase 3 and DAPI positive cells were counted using the cell counter plugin of the Image J software (National Institute of Health (NIH), Bethesda, USA). At least 2000 cells were counted per liver. The proliferation and apoptosis index was calculated by counting the number of Ki67 or active caspase 3 positive cells, respectively, divided by the number of DAPI positive nuclei (total number of cells).

### 2.5. Western Blot Analysis

Livers and liver tumors were dissected and snap frozen in liquid nitrogen. Snap frozen tissues were homogenized in Cell Lysis Buffer (Cell Signaling Technology) supplemented with complete protease inhibitors (Roche). Expression of AFP was analyzed by western blot analysis using standard procedures. Rabbit anti-AFP (1/1000, Abcam) and rabbit anti alpha-actin (1/1000) diluted in PBS with 0.5% (w/v) nonfat dry milk and 0.2% (v/v) Triton X100 were used as primary antibodies. Detection of proteins was carried out with the ECL method using the Western Lightning enhanced luminol-based chemiluminescence HRP substrate (Perkin Elmer).

### 2.6. Statistical Analysis

Data are represented as mean ± SEM and significance is shown on graphs as *P* < 0.05^*∗*^, *P* < 0.01^*∗∗*^, or *P* < 0.001^*∗∗∗*^. Student's* t*-tests or two-way ANOVA tests were used as indicated in the figure legends.

## 3. Results

### 3.1. Expression Levels of the Constitutively Secreted PCs in Liver Tumors of ASV-B Mice

In this study, the role of the PCs and more specifically the role of* Furin* were investigated in a mouse model for HCC. Therefore, the ASV-B mice were used as a well-characterized tumor model for HCC [30]. These mice express the SV40 large T antigen under the control of the anti-thrombin III promoter and mimic the progression of human HCC. Like in humans, these mice show an age dependent progression from hyperplasia/dysplasia (4–8 weeks) to nodular adenoma (12 weeks) and finally carcinoma (>16 weeks). In the studies presented here, 5-, 13-, and 19-week-old mice were investigated, corresponding to the different stages of the tumor progression.

**Figure 1:**
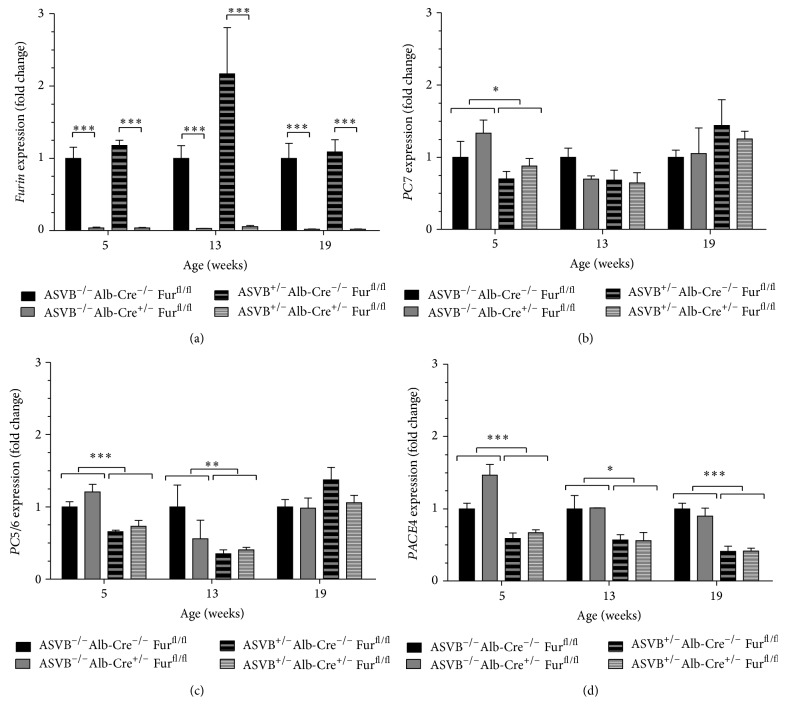
Expression levels of the constitutively secreted proprotein convertases in the livers and liver tumors. (a) The expression levels of* Furin* in* Furin *wild type mice did not change significantly in the liver tumors compared to those in normal livers at every time point investigated. (b) Expression of* PC7* was significantly reduced in the liver tumors of 5-week-old mice, but not at later time points. (c) The expression of* PC5/6* was significantly reduced in the liver tumors of 5-week-old and 13-week-old mice. (d) The expression levels of PACE4 were significantly reduced in the tumors at all time points investigated. (a)* Furin* expression was nearly completely ablated in the livers of the* Furin* KO mice with and without the tumors as shown by qRT-PCR at the different time points investigated. (b, c, d)* Furin* inactivation in the liver and liver tumors did not significantly alter the expression levels of other PCs. Two-way ANOVA was used to determine statistical significance. Data are shown as fold induction ± SEM relative to ASVB^−/−^ Alb-Cre^−/−^  Fur^fl/fl^ mice (*n* = 3–11).

Since it has been described that proprotein convertases are often differentially expressed in tumors, the expression levels of the constitutively secreted proprotein convertases (Furin, PC5/6, PC7, and PACE4) were analyzed in the liver tumors of ASV-B mice. The expression levels of* Furin* in ASVB^+/−^ Alb-Cre^−/−^  Fur^fl/fl^ mice did not change significantly in the liver tumors compared to those in normal livers at every time point investigated (Figure 1(a)). In contrast, the expression of PC7 was significantly reduced in the liver tumors of 5-week-old mice (Figure 1(b)), the expression of* PC5/6* was significantly reduced in the liver tumors of 5-week-old and 13-week-old mice (Figure 1(c)), and the expression levels of PACE4 were significantly reduced in the liver tumors at all time points investigated (Figure 1(d)).

### 3.2. Conditional Inactivation of* Furin* in the Liver of ASV-B Mice Resulted in an Early Tumor Onset

**Figure 2:**
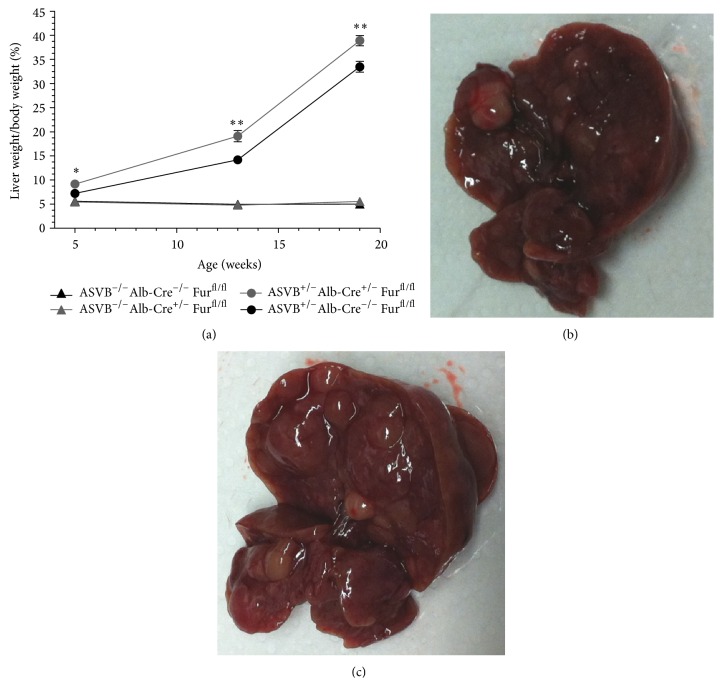
Conditional inactivation of* Furin* in the livers of ASV-B mice resulted in an increased tumor mass. (a) Conditional inactivation of Furin in the livers of ASV-B mice had a significant impact on the tumorigenic process. Already after 5 weeks the ratio of the tumor weight and the total body weight was significantly increased (27%) in the* Furin* KO mice. Student's* t*-test was used to determine statistical significance (*n* = 3–14). (b) A representative photograph of 19-week-old ASVB^+/−^ Alb-Cre^−/−^  Fur^fl/fl^ mice. (c) A representative photograph of 19-week-old ASVB^+/−^ Alb-Cre^+/−^  Fur^fl/fl^ mice.

To explore the* in vivo* role of* Furin* in hepatocellular carcinoma, the* Furin *gene was inactivated in the hepatocytes of ASV-B mice using the Cre/LoxP system.* Furin *was genetically ablated in the HCC tumors and in the livers of littermate control mice via intercrossing with Alb-Cre mice as described in materials and methods. This resulted in a nearly complete ablation of* Furin* expression (>95%) in the livers of the* Furin* KO mice with and without the tumors as shown by qRT-PCR at the different time points investigated (Figure 1(a)). No compensatory upregulation was observed for any of the PCs in* Furin* knockout tissue (Figure 1).* Furin* inactivation had a significant impact on the tumorigenic process. Already after 5 weeks, the ratio of the tumor weight relative to the total body weight was significantly increased by 27% in the* Furin* KO as compared to Furin wild type mice (Figure 2(a)). No significant differences in total body weight were observed between genotypes. In the ASV-B mouse model, the SV40 large T-antigen is expressed in all hepatocytes. This leads to hyperplasia and tumor formation in the whole liver. In contrast to human HCC, the tumor mass is thus not surrounded by normal liver. Photographs of the liver tumors of 19-week-old animals are provided in Figures 2(b) and 2(c). Upon histological analysis of the tumors, no differences could be observed between the tumors for* Furin* wild type mice and* Furin* knockout mice (data not shown).

### 3.3. The Enlarged Tumor Mass after Genetic Ablation of* Furin* Is Caused by an Increased Proliferation

**Figure 3:**
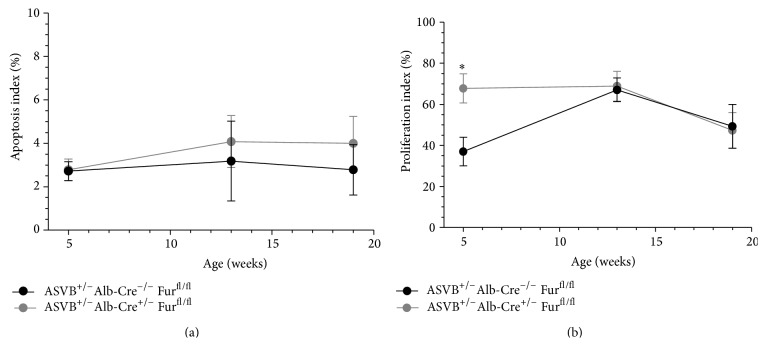
The increased tumor mass after genetic ablation of* Furin* in the liver can be explained by a decreased apoptosis and/or an increased proliferation of the hepatocytes. (a) The apoptosis index in the liver tumors of* Furin* knockout mice was similar to that of wild type littermates at every time point investigated. (b) The hepatocyte proliferation index was significantly increased in the liver tumors of 5-week-old* Furin* knockout mice as compared to those in the liver tumors of littermate* Furin* wild type mice. This difference could not be observed in older mice. Increased proliferation is thus at least one of the mechanisms that can explain the increased tumor mass after genetic ablation of* Furin* in the liver tumors. Student's* t*-test was used to determine statistical significance (*n* = 3-4).

The increased tumor mass after genetic ablation of* Furin* in the liver could be explained by a decreased apoptosis and/or an increased proliferation of the hepatocytes. The apoptosis index was determined at the different time points investigated as described in Materials and Methods. The apoptosis index in the liver tumors of* Furin* knockout mice was similar to that of wild type littermates at every time point investigated (Figure 3(a)). However, the hepatocyte proliferation index was significantly increased in the liver tumors of 5-week-old* Furin *knockout mice as compared to those in the liver tumors of littermate* Furin* wild type mice (Figure 3(b)). This difference was not observed in older mice. Therefore, increased proliferation is at least one of the mechanisms that can explain the early tumor onset after genetic ablation of* Furin* in the liver tumors.

### 3.4. HCC Biomarkers were Expressed at Similar Levels in* Furin *Wild Type and Conditional* Furin* Knockout Mice

**Figure 4:**
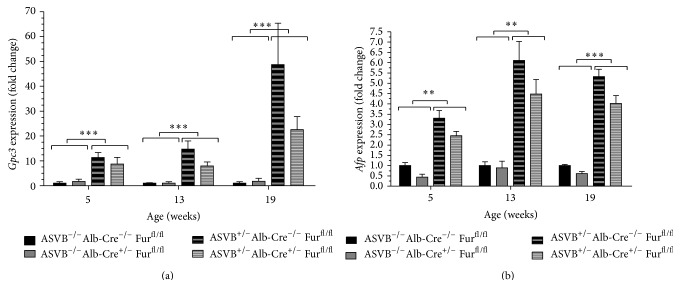
Expression of biomarkers for human HCC is not altered upon genetic ablation of Furin in the liver. The expression levels of* Glypican 3* (*Gpc3*) (a) and* alpha-fetoprotein* (*Afp*) (b) were significantly increased in the liver tumors compared to those in normal livers at every time point investigated as assessed by qRT-PCR. However, the expression levels of* Gpc3* and* Afp* were similar in tumors of* Furin* knockout and* Furin* wild type mice as assessed by qRT-PCR. Two-way ANOVA was used to determine statistical significance. Data are represented as fold induction ± SEM relative to ASVB^−/−^ Alb-Cre^−/−^  Fur^fl/fl^ mice (*n* = 3–5).

**Figure 5:**
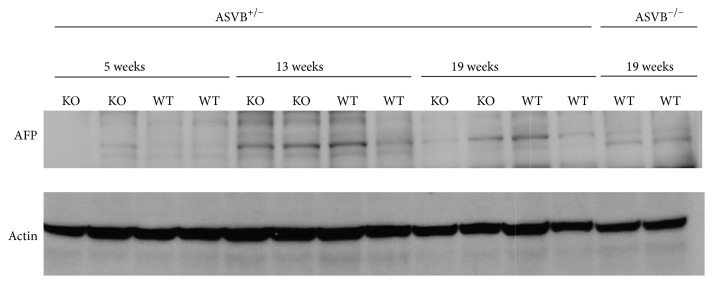
Expression of AFP is not altered upon genetic ablation of* Furin* in the liver. The expression of AFP was increased in the liver tumors of 13- and 19-week-old mice compared to that in normal livers as assessed by western blot analysis. However, the expression of AFP was similar in tumors of* Furin* knockout and* Furin* wild type mice.

We subsequently analyzed the expression pattern of two biomarkers which are frequently used to detect HCC in humans, in the liver. The ASV-B transgene significantly upregulated* Glypican 3* (*Gpc3*) (5 weeks: *P* = 0.000203, 13 weeks: *P* = 0.000002, 19 weeks: *P* < 0.000001) and* alpha-fetoprotein* (*Afp*) (5 weeks: *P* = 0.001136, 13 weeks: *P* = 0.001454, 19 weeks: *P* = 0.000001) expression (Figure 4). Therefore, the expression levels of these genes were significantly increased in the liver tumors as compared to those in normal livers as demonstrated by qRT-PCR. Nevertheless, there was no significant effect of the* Furin* inactivation on the expression levels of* Gpc3* (5 weeks: *P* = 0.72, 13 weeks: *P* = 0.29, 19 weeks: *P* = 0.81) and* Afp *(5 weeks: *P* = 0.12, 13 weeks: *P* = 0.61, 19 weeks: *P* = 0.11) in the liver tumors by qRT-PCR. Likewise, also at the protein level the expression of AFP increased in the tumors of 13- and 19-week-old mice and the expression of AFP was similar in tumors of* Furin* knockout and* Furin* wild type mice (Figure 5).

## 4. Discussion

FURIN inhibition has been proposed as a therapeutic strategy for several tumor types [4, 14, 16]. However, so far few studies reported about the role of* Furin* in HCC [27, 28]. Here we show in an* in vivo* model for HCC that genetic ablation of Furin does not provide therapeutic benefit. In contrast, we observed enhanced early-onset tumor development. The ASV-B transgenic mouse model used here to investigate the role of* Furin* in HCC [30] mimics human HCC quite well [30]. Like in human HCC, the tumor progresses from a hyperplasia/dysplasia stage to a nodular adenoma and finally a carcinoma stage. We demonstrated that the expression of several biomarkers used to detect HCC in humans, such as* Gpc3* and* Afp,* is also upregulated in this tumor model, providing evidence at the molecular level that the tumorigenic process resembles human HCC. Despite the similarities with the human tumors, there are also some differences. The SV40 large T antigen is expressed under the control of the anti-thrombin III promoter in almost all hepatocytes, leading to severe hyperplasia of the liver tissue and finally a huge multifocal tumor mass (up to almost 40% of the total body weight), which is not surrounded by normal liver tissue. In contrast, in humans the tumor arises from a single or few progenitor cells and is surrounded by normal liver tissue. Nevertheless, given the histological and molecular similarities with human HCC, this model is suitable to investigate the role of* Furin* in HCC development and progression. Therefore,* Furin *was genetically ablated in the hepatocytes of the ASV-B mice. This resulted in increased hepatocyte proliferation and increased tumor mass already at 5 weeks. This is in agreement with data obtained from HCC patients where* Furin* overexpression predicts a better postoperative disease-free survival and suggests that* Furin* inhibition in the liver will not be a good therapeutic strategy for liver cancer [28]. Thus, depending on the tumor type,* Furin *inhibition might be advantageous as an anticancer strategy, such as in pleomorphic adenomas of the salivary glands, or disadvantageous. These tumor type dependent differences in the impact of* Furin* ablation on the tumorigenic process could be explained by different molecular mechanisms underlying the tumor formation. For example, one of the major signaling pathways involved in the tumorigenic process of* PLAG1*-induced pleomorphic adenomas of the salivary gland is the IGF signaling pathway. Indeed,* Igf2* isa direct target gene of the transcription factor PLAG1 [33], which plays an important role in the tumorigenic process, since genetic ablation of* Igf2* in the* PLAG1* transgenic mouse model significantly delayed the tumor formation [18]. We previously demonstrated that genetic ablation of* Furin* in salivary gland tumors interfered with IGF signaling since it resulted in an impaired processing of the IGF1R [16]. This might, at least in part, explain the delay in the tumor formation upon* Furin *inactivation in this tumor type. Given the wide range of substrates of FURIN, the overall result is probably a compound phenotype caused by complete or partial inhibition of cleavage of a number of substrates.

In contrast, in the ASV-B mice, the transforming activity of the SV40 large T antigen is largely due to its inactivation of the retinoblastoma proteins, which induces cell cycle entry and blocks the p53 tumor suppressor [34]. So far no FURIN substrates have been identified downstream of this signaling cascade. Nevertheless, the lack of* Furin* in hepatocytes in this mouse model enhances the HCC tumorigenic process. However, the FURIN substrate(s) leading to the early tumor onset is currently unknown and will be part of further investigations.

## 5. Conclusions

It has previously been shown that FURIN inhibition reduces tumorigenesis in various human cancer cells, suggesting that* Furin* inhibition might be a good therapeutic strategy for cancer [12]. However, here we demonstrate that the impact of Furin inactivation on the tumorigenic process depends on the tumor type.* Furin* inhibition in a mouse model for HCC leads to early tumor onset with significantly larger tumors due to increased hepatocyte proliferation. Therefore,* Furin *inhibition will not be a good therapeutic strategy for HCC and might even enhance tumor formation.

## Supplementary Material

Supplementary Table 1: Primer sequences used for the transcript detection by qRT-PCR.
