# Supplementary material for: Liver-Specific Inactivation of the Proprotein Convertase FURIN Leads to Increased Hepatocellular Carcinoma Growth
Source: Biomed Res Int. 2015 Jun 8;2015:148651. doi: 10.1155/2015/148651 (PMC4475760; doi:10.1155/2015/148651)
Supplement: Supplementary file 1 — Supplementary Table 1: Primer sequences used for the transcript detection by qRT-PCR. [file 148651.f1.docx]

**Supplementary Table 1**: Primer sequences used for the transcript detection by qRT-PCR.

| Gene | Sequence |
| --- | --- |
| *Gapdh*  *Furin*  *PC7*  *PC5*  *PACE4*  *Gpc3*  *Afp* | Forward: 5’ CCCCAATGTGTCCGTCGTG 3’  Reverse: 5’ GCCTGCTTCACCACCTTCT 3’  Forward: 5’ CAGAAGCATGGCTTCCACAAC 3’  Reverse: 5’ TGTCACTGCTCTGTGCCAGAA 3’  Forward: 5’ CCACCCTGATGAGGAGAATG 3’  Reverse: 5’ GCCACAGCCTCCATACTGTC 3’  Forward: 5’ GATGACGGCATCGAGAGAAC 3’  Reverse: 5’ TCATTGCTTGCATCATAACGA 3’  Forward: 5’ AGCGTGGCTACACAGGAAAG 3’  Reverse: 5’ TATCTCGGGGATGGGTCATA 3’  Forward: 5’ ATTGGAAGCTCTGGTGACG 3’  Reverse: 5’ TCCACATCCAGATCATAGGC 3’  Forward: 5’ GCCCTACAGACCATGAAACAAG 3’  Reverse: 5’ GTGAAACAGACTTCCTGGTCCT 3’ |
